# Development of a Novel Nordic Hamstring Exercise Performance Test Device: A Reliability and Intervention Study

**DOI:** 10.3390/sports10020026

**Published:** 2022-02-21

**Authors:** Jesper Augustsson, Sofia Ryman Augustsson

**Affiliations:** Department of Sport Science, Faculty of Social Sciences, Linnaeus University, 39182 Kalmar, Sweden; sofia.rymanaugustsson@lnu.se

**Keywords:** soccer, hamstring muscles, soft tissue injuries, resistance training, athletic performance

## Abstract

There is evidence that a knee flexor exercise, the Nordic hamstring exercise (NHE), prevents hamstring injuries. The purpose of this study was therefore to develop, and to determine the reliability of, a novel NHE test device and, further, to determine the effectiveness of a 10-week low volume NHE program on NHE performance. Twenty female football (soccer) players, aged 16–30 years, participated in this study. From a kneeling position on the device, with the ankles secured under a heavy lifting sling, participants leaned forward in a controlled manner as far as possible (eccentric phase) and then returned to the starting position (concentric phase). A tape measure documented the forward distance achieved by the participants in cm. Participants completed three separate occasions to evaluate test-retest reliability. Additionally, 14 players performed a low volume (1 set of 5 repetitions) NHE program once weekly for 10 weeks. No significant test-retest differences in NHE performance were observed. The intra-class correlation coefficient was 0.95 and the coefficient of variation was 3.54% between tests. Mean improvement in the NHE performance test by the players following training was 22% (8.7 cm), *p* = 0.005. Our test device reliably measured NHE performance and is easy to perform in any setting. Further, NHE performance was improved by a 10-week low volume NHE program. This suggests that even a small dose (1 set of 5 repetitions once weekly) of the NHE may enhance NHE performance.

## 1. Introduction

Hamstring muscle injury (and reinjury) is the most common muscle injury in many sports including football [1,2,3] and track and field [4]. Acute hamstring injury can be divided into at least two categories: high-speed running or stretching-type injuries [5]. The most common hamstring injury type typically occurs during high-speed running [6]. In several intervention studies researchers have used an eccentric strengthening protocol of the knee flexors that have successfully reduced the incidence rates of hamstring injuries [7,8,9]. Further, weak eccentric hamstrings strength has been established as an important modifiable risk factor for hamstring injuries [10]. The most common exercise performed in injury prevention programs is the Nordic hamstring exercise (NHE) [11]. The predominant way the NHE has been performed in research is eccentrically (where athletes lean forward and then fall to the floor in a controlled fashion, catching themselves with their hands). In sports, however, it is relatively common for strength and conditioning coaches and physiotherapists alike to have the athletes perform the exercise eccentrically-concentrically. Further, some elite athletes can perform a full NHE to the floor and back without a break point, in other words an eccentric-concentric NHE. As the NHE has recently been recognized to decrease the risk of hamstring injuries among athletes by half [12], it could be said that a large amount of scientific evidence now supports the use of this exercise to prevent hamstring injuries. Yet it is an elusive exercise, as there is no consensus on how to use it effectively in clinical practice when it comes to e.g., dosing, and further, today there is no simple and practical field-test to evaluate NHE performance. Currently, the gold standard to assess the effect of a NHE program on knee flexor strength is isokinetic dynamometry [13]; however, the validity of this method for predicting hamstring strain risk has been questioned [14]. Moreover, isokinetic dynamometry is expensive and rarely used outside the laboratory setting. Handheld dynamometry is an assessment tool used in the clinic, in research and by strength professionals. Research has shown, however, that this test may have drawbacks when it comes to collecting reliable and valid data [15,16]. More recently, several devices for the assessment of hamstring strength have been developed [17,18,19]. Typically, these devices use load cells where force data are transferred to a personal computer through a recording unit. Subsequently, the data are analyzed with custom data software. Alternative testing methods, which simply use the NHE itself to the dynamometry-based hamstring assessment tools, are currently lacking.

Therefore, the purpose of this study was to develop, and to determine the reliability of, a novel, simple and easy to use NHE test device. In addition, this study aimed to determine the effectiveness of a low-volume NHE intervention on NHE performance in female football (soccer) players.

## 2. Materials and Methods

### 2.1. Participants

Twenty female footballs players were recruited to the study. Coaches from two female football teams, from the second and fourth highest division in Sweden respectively, were contacted by telephone and the participants were introduced to the study during a visit at one of their practice sessions. The inclusion criteria included being a female athlete aged 16–30 years, participating and competing in football. Table 1 reports the characteristics of participants. The exclusion criteria were knee, hip or back injury prior to testing and training within six months of the study. Prior to inclusion, subjects were informed of the benefits and risks of participation before providing written informed consent.

### 2.2. Procedures

The same investigator collected the reliability NHE performance data on the participants at three separate sessions on different days, five to seven days apart. The first session served as a familiarization session and on the following two occasions NHE performance, using the novel NHE test, was determined. Subsequently, one of the teams (14 players) participated in the exercise intervention part of the study in which the participants performed a low volume (1 set of 5 repetitions) NHE program once per week for 10 weeks.

### 2.3. Development of the Test Device

**Figure 1:**
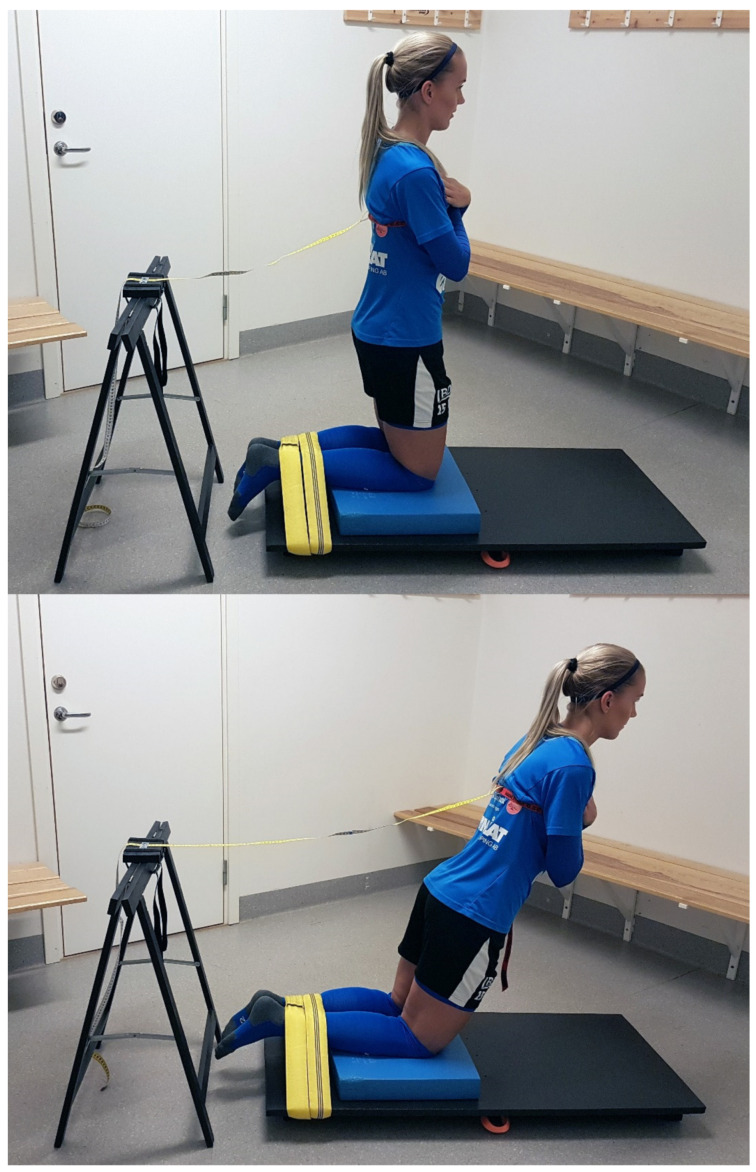
Testing set-up. Upper panel: The participants were placed in a kneeling position over the padded board, ankles secured under a roundsling and arms across the chest. The measuring tape was placed on the participant’s torso via a strap, standardized at a height of 80 cm above the knees. Lower panel: Participants leaned forward in a slow, controlled manner as far as possible (eccentric phase) and then returned to the starting position (concentric phase). The tape measure documented the forward distance achieved by the participants in cm.

The participants were tested for NHE performance using a device that consisted of a balance-pad (Airex, Sins, Switzerland) and a board (55 × 120 × 2 cm) that rested on three transverse wood studs (41 × 4.5 × 7 cm). See Figure 1 for testing set-up. A non-elastic roundsling with an effective working length of 100 cm (GVP Safety, Sweden) was placed around the board, connected by a strap. Further, a trestle (height 73 cm) held a board via two straps onto which a steel band was attached. One end of a soft measuring tape ran under the steel band whereas the other end of the measuring tape was connected to a strap. Lastly, a measuring stick was used to position the measuring tape, via the strap, at 80 cm above knee height on each participant’s torso.

### 2.4. Experimental Set-Up

The participants performed a warm-up consisting of 5-min of ergometer cycling at 50 W and 1 set of 15 repetitions of squats, standing toe raises and hip bridges, respectively. The participants were then placed in a kneeling position over the padded board, ankles secured under the roundsling, the lateral malleolus aligned with the edge of the board and arms across the chest. Five submaximal NHE repetitions (at approximately 50% effort) where the participants leaned forward in a slow, controlled manner (eccentric phase) and then returned to the starting position (concentric phase) were then performed. This was followed by three submaximal, eccentric phase only, NHE repetitions (at approximately 80% effort) where the participants leaned forward and then fell to the floor in a controlled fashion, catching themselves with their hands. The measuring tape was then firmly placed on the participants’ torsos via the strap, standardized at a height of 80 cm above the knees. The trestle, attached to the other end of the measuring tape, was placed behind the participants and adjusted as to take the slack out of the measuring tape. Subsequently, three maximal trials of eccentric-concentric NHE repetitions were performed, where participants leaned forward in a slow, controlled manner as far as possible (eccentric phase) and then returned to the starting position (concentric phase). The maximal NHE trials were separated by a standardized 1-min rest period to allow for recovery and avoid fatigue. The tape measure documented the forward distance achieved by the participants in cm. If participants increased their performance in all three trials, one or two additional NHE repetitions were performed. Verbal commands to the participants were standardized and encouraging. A single investigator, a physical therapist that had more than 25 years of experience of testing and training athletes and patients, monitored all tests and the performance of all trials. Trials were only regarded as successful if the participants held trunk and hips in a neutral position during the NHE repetitions. The tests took place in a secluded room at the respective team facilities five to seven days apart.

### 2.5. Exercise Intervention Protocol

Following the reliability NHE performance part of the study, one of the teams (14 players) performed a low volume (1 set of 5 repetitions) NHE program once per week for 10 weeks. As part of the football practice warm-ups, the players performed five submaximal eccentric-concentric NHE repetitions (at approximately 50% effort) and five submaximal eccentric NHE repetitions (at approximately 80% effort). After these progressive warm-up repetitions, the players performed five maximal eccentric-concentric NHE repetitions. The players performed the NHE on the football field in pairs (i.e., with a partner), where one player assisted by holding the ankles and acting as a counterweight. The way the NHE was performed in the intervention did not differ from the tests. The investigators supervised all training sessions. Verbal commands to the players were standardized and encouraging. Data from players who did not attend at least 70% of the training sessions were not included in the analysis, as recommended by Weatherwax et al. [20]. All players performed the novel NHE test within seven days before and after the 10-week training intervention. No adverse events, in the form of injury, occurred during the 10-week training intervention.

### 2.6. Statistical Analyses

All statistical analyses were performed using IBM SPSS Statistics (version 26, IBM, Armonk, NY, USA) except for the statistical power analysis, where PS—Power and Sample Size Calculation (version 3, W.D. Dupont & W.D. Plummer, Nashville, TN, USA) was used. The results are presented as means with SDs. To estimate the test-retest reliability of the NHE test, intraclass correlation coefficients (ICC) [21] with the two-way random effects model of the measurements with 95% confidence interval (CI) were used. The ICC were classified in the following manner: greater than 0.90, high reliability; between 0.80 to 0.89, good reliability; between 0.70 to 0.79, fair reliability and values less than 0.69, poor reliability [22]. Further, within-subject variation was determined using typical error expressed as a coefficient of variation (CV) [23]. A paired samples t-test was used to detect significant test-retest differences. The significance levels for all analyses were set to *p* < 0.05. The effect size was calculated for determining the magnitude of the training effect, using the following formula: Pre-post effect size = posttest mean − pretest mean/pretest SD [24]. A scale for determining the magnitude of effect size in trained athletes was used that identified <0.25 as representing a trivial effect, 0.25–0.5 a small effect, 0.50–1.0 a moderate effect and 1.0 or greater as a large effect [24]. Statistical power analysis: Based on a hypothesized 20% difference in performance as a result of the NHE intervention, 10 was the estimated number of participants required to achieve a power of 0.90. Twenty percent improvement in performance, as recommended by Grant et al. [25], represents a meaningful clinical difference. This study was designed to recruit a minimum of 14 participants to allow for potential dropout.

## 3. Results

**Figure 2:**
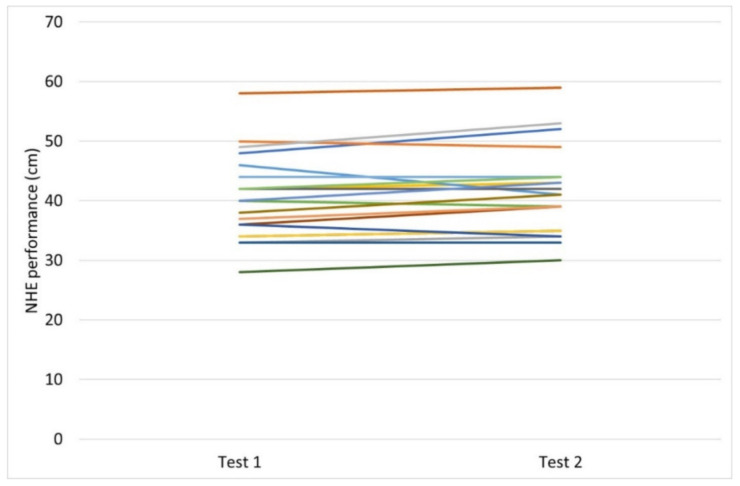
Descriptive data for test-retest differences in NHE performance (cm) in the participants (*n* = 20).

**Figure 3:**
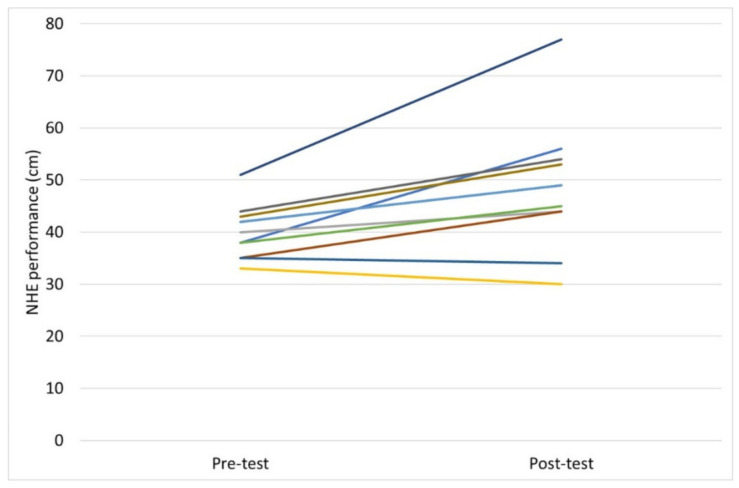
Descriptive data for pre versus post training intervention changes in NHE performance (cm) in the participants (*n* = 11).

No significant test-retest differences were observed in NHE test performance by the participants. Mean ± SD forward distance achieved for test 1 was 40.5 cm ± 7.2 vs. 41.4 cm ± 7.4, for test 2 (*p* = 0.064). The ICC value was 0.95, the CI 0.87–0.98 and the CV 3.54%. NHE test performance significantly improved in the players following 10 weeks of NHE intervention. Mean improvement in NHE test performance for the players (*n* = 11) following training, assessed by the novel NHE test, was 22% (8.7 cm ± 8.1), *p* = 0.005. The effect size was determined to be 1.7, representing a large effect. All players included in the statistical analysis completed at least 70% of the training sessions, with an average attendance of 93%. The figures of descriptive data show test-retest differences (Figure 2) and pre-post intervention changes (Figure 3) in NHE performance, respectively, in the participants.

## 4. Discussion

The main finding of this study was that the novel field-based test device of NHE performance showed high reliability (ICC = 0.95) in female football players. Further, NHE test performance was significantly improved (22%) by a low volume (1 set of 5 repetitions once per week) NHE program performed for 10 weeks.

In recent years, several studies have been published on new devices for the assessment of knee flexor strength [17,18,19]. One of these devices [17] has been used in research [26,27,28] and has since become commercially available, known as the NordBord testing system [29]. Whilst we recognize the importance of these new devices in research and in practice, they are relatively expensive and not an option for many sports teams and athletes. Our device is a practical and easy to perform field-based test that uses the NHE itself to measure NHE performance at a minimal cost and therefore may offer an inexpensive alternative for physical therapists, researchers, strength professionals, sports teams and athletes alike.

To our knowledge, this is the first intervention study investigating the effect of the NHE performed both eccentrically and concentrically. We noted a significant increase in NHE performance (22%) after 10 weeks of training once weekly. This is in contrast to a recent study by Medeiros et al. [30] in which an 8-week NHE program performed eccentrically once weekly did not result in greater hamstring strength of football players. The different results may be explained by several factors. For example, changes in strength in the study by Medeiros et al. [30] was measured through isokinetic dynamometry, a test that may not be sensitive to improvements in isotonic strength [31]. Furthermore, the relationship between hamstring strength recorded isokinetically and NHE strength assessed by the eccentric hamstring strength device developed by Opar et al. [17] was poorly correlated [26]. The low association between these tests implies that they might measure different qualities of muscle strength.

The training volume in the present study was low: 1 set of 5 repetitions per week (not including the warm-up repetitions). In a recent systematic review and meta-analyses [11] on the effect of NHE training volume, it was concluded that low training volumes may be appropriate for athletes since low training volumes can produce large-to-very large improvements in knee flexor strength. A minimal dosage for the NHE has yet to be determined; however, we believe that the 1 set of 5 repetitions per week protocol used in our study is probably at the very end of the low training volume spectrum where changes in strength still occur. We also believe that by increasing the training volume it would be possible to attain larger increases in strength; however, doing so may very well lead to lower compliance in football players [32]. Moreover, in the short term the NHE may detrimentally affect e.g., sprint performance. Recently, it was noted that an NHE session reduced maximal sprint performance in athletes up to 48 h [33]. Therefore, with the aim of keeping compliance and adherence among the athletes at its highest, perhaps a compromise when it comes to training volume is to be recommended.

There are two possible origins of improvements in NHE performance by the players in the intervention part of our study: neural adaptation (a learning effect as a result of strength training in which adaptive changes occur within the nervous system) and muscle hypertrophy. It is widely considered that neural adaptation, rather than hypertrophy, plays the dominant role in increases in performance during the initial phase of strength training [34]. Moreover, it seems as if high-load strength training results in greater neural adaptations than low-load strength training [35]. In the present study, the players performed each NHE with maximal effort/intensity. It is therefore suggested that the increases in NHE performance that were observed in the present study may be explained mainly by neural adaptation (although adaptation in the form of hypertrophy most likely occurred as well).

In studies on the NHE, this exercise is normally performed eccentrically [8,10,11,17,26,27,30]. In contrast, the NHE repetitions were, as mentioned earlier, performed eccentrically-concentrically in our study. This is, however, the way repetitions generally are performed in strength training and testing when it comes to key exercises such as the bench press and barbell squat. Benefits from performing a combined eccentric-concentric muscle action instead of only the eccentric phase of the NHE may include that the total amount of work is higher (a “full” repetition compared with a “half” repetition). Further, the hamstrings act both eccentrically and concentrically during the test and thus arguably more closely mimic sports movements such as running and jumping.

There are some of limitations to the current study. The generalizability of the present study is limited to female football players. Further studies on other athletic populations are therefore desirable. In addition, the question of the validity of the novel measurement should be addressed. Our test device assesses NHE performance rather than knee flexor strength. Nonetheless, to validate against the gold standard for measuring knee flexor strength—isokinetic dynamometry—is something for future research. Further, the second part of the study in which the players performed the NHE intervention lacked a control group. However, the players can be considered as their own controls because they were tested twice (with no significant difference between tests) before the intervention [36]. Finally, to minimize the risk of injury the players performed a warm-up that included submaximal repetitions of NHE before both testing and training. However, we believe the improvements among the players originate from the maximal NHE repetitions and the effect of the warm-up on NHE test performance to be negligible.

## 5. Conclusions

The novel test device reliably measured NHE performance in female football players. With the help of this portable test device, planning and monitoring of various NHE interventions is possible in a quick, easy, inexpensive and reliable way. Further, NHE performance was improved by a low volume 10-week NHE program. This suggests that even a small dose (1 set of 5 repetitions once per week) of the NHE may improve NHE performance, but optimal dosing is still unclear [11].

## Figures and Tables

**Table 1:** Characteristics of participants (*n* = 20).

Characteristics	Mean ± SD
Age, year	20 ± 4
Height, m	1.69 ± 0.06
Weight, kg	63 ± 6
Football practice, hours per week	5.4 ± 1

## Data Availability

The data presented in this study are available on request from the corresponding author.
